# Estimates of incidence and mortality of cervical cancer in 2018: a worldwide analysis

**DOI:** 10.1016/S2214-109X(19)30482-6

**Published:** 2019-12-04

**Authors:** Marc Arbyn, Elisabete Weiderpass, Laia Bruni, Silvia de Sanjosé, Mona Saraiya, Jacques Ferlay, Freddie Bray

**Affiliations:** a Unit of Cancer Epidemiology, Belgian Cancer Centre, Sciensano, Brussels, Belgium; b International Agency for Research on Cancer, Lyon, France; c Consortium for Biomedical Research in Epidemiology and Public Health, Barcelona, Spain; d Unit of Infections and Cancer, Catalonian Institute of Oncology, Barcelona, Spain; e PATH, Seattle, WA, USA; f Centers for Disease Control and Prevention, Division of Cancer Prevention and Control, Atlanta, GA, USA

## Abstract

**Background:**

The knowledge that persistent human papillomavirus (HPV) infection is the main cause of cervical cancer has resulted in the development of prophylactic vaccines to prevent HPV infection and HPV assays that detect nucleic acids of the virus. WHO has launched a Global Initiative to scale up preventive, screening, and treatment interventions to eliminate cervical cancer as a public health problem during the 21st century. Therefore, our study aimed to assess the existing burden of cervical cancer as a baseline from which to assess the effect of this initiative.

**Methods:**

For this worldwide analysis, we used data of cancer estimates from 185 countries from the Global Cancer Observatory 2018 database. We used a hierarchy of methods dependent on the availability and quality of the source information from population-based cancer registries to estimate incidence of cervical cancer. For estimation of cervical cancer mortality, we used the WHO mortality database. Countries were grouped in 21 subcontinents and were also categorised as high-resource or lower-resource countries, on the basis of their Human Development Index. We calculated the number of cervical cancer cases and deaths in a given country, directly age-standardised incidence and mortality rate of cervical cancer, indirectly standardised incidence ratio and mortality ratio, cumulative incidence and mortality rate, and average age at diagnosis.

**Findings:**

Approximately 570 000 cases of cervical cancer and 311 000 deaths from the disease occurred in 2018. Cervical cancer was the fourth most common cancer in women, ranking after breast cancer (2·1 million cases), colorectal cancer (0·8 million) and lung cancer (0·7 million). The estimated age-standardised incidence of cervical cancer was 13·1 per 100 000 women globally and varied widely among countries, with rates ranging from less than 2 to 75 per 100 000 women. Cervical cancer was the leading cause of cancer-related death in women in eastern, western, middle, and southern Africa. The highest incidence was estimated in Eswatini, with approximately 6·5% of women developing cervical cancer before age 75 years. China and India together contributed more than a third of the global cervical burden, with 106 000 cases in China and 97 000 cases in India, and 48 000 deaths in China and 60 000 deaths in India. Globally, the average age at diagnosis of cervical cancer was 53 years, ranging from 44 years (Vanuatu) to 68 years (Singapore). The global average age at death from cervical cancer was 59 years, ranging from 45 years (Vanuatu) to 76 years (Martinique). Cervical cancer ranked in the top three cancers affecting women younger than 45 years in 146 (79%) of 185 countries assessed.

**Interpretation:**

Cervical cancer continues to be a major public health problem affecting middle-aged women, particularly in less-resourced countries. The global scale-up of HPV vaccination and HPV-based screening—including self-sampling—has potential to make cervical cancer a rare disease in the decades to come. Our study could help shape and monitor the initiative to eliminate cervical cancer as a major public health problem.

**Funding:**

Belgian Foundation Against Cancer, DG Research and Innovation of the European Commission, and The Bill & Melinda Gates Foundation.

## Introduction

10 years ago, cervical cancer ranked as the third most common cancer among women worldwide. However, in 42 low-resource countries, it was the most common cancer in women.^1^ The knowledge that persistent infection with carcinogenic human papillomavirus (HPV) types is the main cause in triggering the development of cervical cancer has opened new pathways for primary and secondary prevention.^2^ The implementation of both methods of prevention can make cervical cancer occurrence and death largely avoidable.

Consistent evidence indicates that the licensed bivalent and quadrivalent HPV vaccines containing HPV16 and HPV18 antigens protect with high efficacy against infection and precancerous cervical lesions associated with these types when individuals are not yet exposed.^3^ Both types jointly cause 70–75% of all cervical cancers and 40–60% of its precursors.^4^, ^5^ In the past few years, a nonavalent vaccine has also been licenced, which protects against seven carcinogenic HPV types that, together, cause approximately 90% of cervical cancers.^6^

**Research in context**

**Evidence before this study**

The Global Cancer Observatory (GLOBOCAN) is a regularly-updated database, compiled by the International Agency for Research on Cancer, of global estimates of incidence and mortality rate for 36 cancers and for all cancers combined. For compiling the estimates, recorded data of high quality from national or subnational cancer registry sources are used where possible, otherwise the best available local sources are used in their absence. Previous GLOBOCAN estimates for 2008 indicated that approximately 530 000 cervical cancer cases and 275 000 deaths had occurred worldwide, with 85% of cases occurring in less developed countries. The estimated annual age-standardised incidence rate (ASIR) was 15 per 100 000 women globally and ranged from less than 1 to 56 per 100 000. Cervical cancer was the leading cause of cancer-related death among women in sub-Saharan Africa, central America, south-central Asia, and Melanesia.

**Added value of this study**

Our study provides updated estimates of the cervical cancer burden 10 years after the 2008 GLOBOCAN publication. In 2018, cervical cancer remained a major public health problem, ranking as the fourth most common cause of cancer incidence and mortality in women worldwide. Cervical cancer was the main cause of cancer-related deaths in women in eastern, middle, southern, and western Africa. We observed a significant positive correlation between national ASIRs and corresponding estimates of human papillomavirus (HPV) prevalence. The 2018 estimates presented here provide a baseline to measure the future achievements regarding the ambitious rollout of the WHO Global Initiative to eliminate cervical cancer as a public health problem in every country of the world.

**Implications of all the available evidence**

Today, new tools of primary prevention (prophylactic HPV vaccination) and secondary prevention (screening with validated HPV assays and treatment of cervical precancerous lesions) have been shown to be effective. The ambition of WHO is to reduce the ASIR of cervical cancer to less than 4 per 100 000 women worldwide by vaccinating 90% of all girls by age 15 years, screening 70% of women twice in the age range of 35–45 years, and treating at least 90% of all precancerous lesions detected during screening. Modellers have estimated that this goal might be reached within a few decades in high-resource countries, but might take until the end of the 21st century for the goal to be reached in the lowest-resource countries. Nevertheless, the return of investment will be highest in low-resource countries; for example, using the cumulative incidence estimates from GLOBOCAN and assuming 70% HPV vaccination effectiveness, we can calculate that only 20 girls would need to be vaccinated in eSwatini (the country with the highest estimated incidence) to avoid one case of cervical cancer, whereas the equivalent number needed would be 238 girls in the USA. Ensuring the availability of recorded data of good quality from population-based cancer registries will be essential for monitoring local progress towards the cervical cancer elimination goal.

The treatment of precancerous lesions detected by microscopic inspection of cells scraped from the cervix has been the paradigm of secondary prevention of cervical cancer for half a century.^7^ Although cytological screening has undoubtedly led to a major decline in cervical cancer burden in several resource-rich countries, the method might have reached its limits, with reports from several countries with longstanding high-quality Pap smear-based programmes indicating that trends have either stabilised or began to rise.^8^ Meta-analyses and pooled analyses of randomised trials have shown that screening with HPV tests protects better against future cervical precancerous lesions and invasive cancers than screening by cytology^9^, ^10^ and, therefore, virological screening programmes are becoming increasingly recommended.^11^, ^12^

Given the availability of these new preventive tools, public health experts are challenged to define comprehensive integrated strategies that combine HPV vaccination and cervical cancer screening that fit the target populations within the limits of cost-effectiveness. In 2018, in a greatly changing preventive landscape, the WHO Director-General launched an ambitious call to all nations of the world to mobilise resources to make an end to suffering from cervical cancer.^13^

Now more than ever, effective cervical cancer control planning requires access to accurate statistics. According to WHO, one of the fundamental steps in the action plan for non-communicable diseases is to establish a high-quality surveillance and monitoring system that provides, as a minimum standard, reliable population-based statistics data on the major non-communicable diseases.^14^

Using the 2018 estimates of the worldwide cancer burden compiled by the International Agency for Research on Cancer (IARC) on the basis of available cancer registry and vital statistics data, we describe in this study the existing patterns of cervical cancer incidence and mortality rate alongside HPV prevalence data, thus allowing a comprehensive baseline assessment of the global cervical cancer burden.^15^

## Methods

### Study design and data sources

We extracted the estimated number of cases of and deaths from cancer of the cervix uteri (International Classification of Diseases tenth edition [ICD-10] code C53) in 185 countries in 2018 from the Global Cancer Observatory (GLOBOCAN) 2018 database, as published by the IARC.^16^, ^17^ Data were aggregated by 5-year age groups, except for the oldest age group comprising women aged 85 years or older. In this study, the 5-year age groups from 15 years to 44 years were merged to assess the burden of cervical cancer in younger women, particularly because few deaths in this age range are classified as uterine cancer not otherwise specified (ICD-10 code C55).^18^, ^19^ A Strengthening the Reporting of Observational Studies in Epidemiology statement, containing the checklist of items to be included in reports of observational studies, is provided in the appendix (pp 15–20).

Data sources and methods of estimation for incidence and mortality rate have been described in detail elsewhere.^17^ Briefly, for estimation of incidence, we applied a hierarchy of methods that were dependent on the availability and quality of the source information from population-based cancer registries; methods ranged from a short-term extrapolation of high-quality recorded national incidences through short-term prediction models^20^ to the use of observed rates from one or more neighbouring countries in the same region in the complete absence of recorded information.

For the estimation of mortality rates, we used the WHO mortality database as a source for the number of deaths caused by cancer where available, with the figures adjusted for incomplete registration and corrected for ill-defined causes of death. Studying cervical cancer mortality is particularly difficult because the certified cause of death often does not indicate the anatomical origin (cervix [CVX] or corpus uteri [CRP]) with sufficient precision, but rather the death is classified as death from uterine cancer, not otherwise specified (NOS). In GLOBOCAN 2018, when the proportion of NOS deaths was less than 25% of all uterine cancer deaths, the corrected incidence of cervical cancer deaths (corCVX_i_) was computed by use of the following reallocation rule:^18^

$${\text{corCVX}}_{\text{i}}={\text{CVX}}_{\text{i}}+\text{NOSi}*{\text{CVX}}_{\text{i}}/({\text{CVX}}_{\text{i}}+{\text{CRP}}_{\text{i}})$$

For some countries with reliable national cancer registries and survival statistics, we estimated corCVX_i_ from age-specific incidence and the 5-year relative survival probability.^17^ No data were available to allow adjustment for hysterectomy.

### Stratification by geographical region and human development

Countries were grouped in 21 subcontinents as defined by the UN except for Cyprus, which was reallocated to southern Europe.^18^ In this study, Micronesia and Polynesia were aggregated to comprise one subcontinental region. Countries were categorised by the Human Development Index (HDI), a composite index of life expectancy, education, and per-capita income indicators developed by the UN Development Programme^21^ (appendix p 1) that ranks countries into four tiers of human development (low, medium, high, and very high). By use of the HDI estimates for 2016, countries within the highest of the four tiers are interchangeably denoted as the highest-resource countries and countries in the remaining three tiers are denoted as lower-resource countries.

### Statistical analysis

We calculated the number of cervical cancer cases and deaths in a given country by applying the estimated age-specific and sex-specific rates for 2018 to the corresponding population strata. We calculated the directly age-standardised incidence rate (ASIR) and age-standardised mortality rate (ASMR) using the world standard population.^22^ We derived the indirectly standardised incidence ratio (SIR) and mortality ratio (SMR) from the ratio of

∑Oi/∑Ei

where O_i_ corresponds to the estimated number of cases or deaths and E_i_ corresponds to their expected number, being the product of

$${\text{a}}_{\text{wi}}*{\text{N}}_{\text{ci}}$$

(world age-specific rates multiplied by the number of women in the corresponding age stratum [i] of each country [c]). We computed the cumulative rates by summing the products of the age-specific rates (a_i_) multiplied by the width of the corresponding age groups (ΔT_i_) up to age 74 years.^23^

$$\text{CR}=\sum {\text{a}}_{\text{i}}*\Delta {\text{T}}_{\text{i}}$$

We computed the average age at diagnosis as the weighted mean age using the mid-age of each 5-year age group and 90 years for women aged 85 years or older.

The geographical distribution of the age-standardised incidence and mortality rate per 100 000 women by country is displayed in choropleth world maps, using categories of ascending rate groupings as used in earlier publications^1^, ^24^ to allow comparisons.

### Role of the funding source

The funders of the study had no role in study design, data collection, data analysis, data interpretation, or writing of the report. The corresponding author had full access to all the data in the study and had final responsibility for the decision to submit for publication.

## Results

In 2018, approximately 570 000 women developed cervical cancer and 311 000 women died from it, corresponding to an all-ages ASIR of 13·1 per 100 000 women-years and ASMR of 6·9 per 100 000 (table). Worldwide, cervical cancer was the fourth most common cancer among women, after breast cancer (2·09 million cases), colorectal cancer (0·79 million), and lung cancer (0·73 million); and it was also the fourth leading cause of cancer death among women, after breast (627 000 deaths), lung (576 000) and colorectal (387 000) cancers. Approximately 84% of all cervical cancers and 88% of all deaths caused by cervical cancer occurred in lower-resource countries (ie, those with HDI <0·80), of which 1·8% of women were diagnosed with and 1·3% died from the disease before age 75 years, in the absence of competing causes of death. By contrast, in the highest-resource countries, the cumulative rates of cervical cancer incidence and mortality were two to four times lower than those in lower-resource countries. The ASIR and ASMR increased with decreasing level of HDI, from an ASIR of 9·6 per 100 000 women and ASMR of 3·0 per 100 000 in countries in the very high HDI tier to an ASIR of 26·7 per 100 000 and ASMR of 20·0 per 100 000 in countries in the low HDI tier (appendix p 2).

**Table:** Burden of cervical cancer incidence and mortality in 2018 worldwide and by the four-tier HDI and by sub-continent

		**Total female population (millions)**	**Number of cases**	**ASIR (per 100 000 women)**	**SIR**	CIR*	**Proportion of all cancers**	**Rank (all ages)**	**Rank (15–44 years)**	**Number of deaths**	**ASMR (per 100 000 women)**	**SMR**	CMR*	**Proportion of all cancers**	**Rank (all ages)**	**Rank (15–44 years)**
World		3782·1	569 847	13·1	100	1·4%	6·9%	4	2	311 365	6·9	100	0·8%	7·5%	4	2
HDI level																
	Very high	680·2	90 032	9·6	67	0·9%	2·8%	12	3	36 305	3·0	44	0·3%	2·6%	12	2
	High	1211·9	180 597	11·1	85	1·1%	6·1%	6	3	85 296	4·9	73	0·5%	5·4%	7	2
	Medium	1339·8	204 130	15·7	118	1·7%	14·4%	2	2	122 097	9·6	139	1·1%	14·3%	2	2
	Low	536·7	93 285	26·7	194	3·0%	17·7%	2	2	66 643	20·0	283	2·4%	22·4%	1	2
Subcontinents																
	Eastern Africa	218·4	52 633	40·1	289	4·4%	26·5%	1	1	37 017	30·0	425	3·5%	27·5%	1	1
	Middle Africa	84·6	12 635	26·8	188	3·1%	23·6%	2	2	9418	21·1	292	2·5%	25·1%	1	2
	Northern Africa	118·3	7652	7·2	52	0·8%	5·2%	4	5	5243	5·1	71	0·6%	6·5%	3	7
	Southern Africa	33·6	14 409	43·1	338	4·3%	23·4%	2	1	6480	20·0	297	2·1%	20·7%	1	1
	Western Africa	189·7	31 955	29·6	199	3·5%	23·3%	2	2	23 529	23·0	309	2·8%	26·6%	1	2
	Caribbean	22·3	4200	15·5	121	1·6%	8·1%	4	2	2464	8·5	127	0·9%	8·7%	4	2
	Central America	90·5	12 406	13·0	101	1·3%	9·1%	2	3	6619	7·0	104	0·8%	10·9%	2	2
	South America	217	39 581	15·2	118	1·6%	7·7%	3	2	19 235	7·1	106	0·8%	8·2%	4	2
	Northern America	183·7	15 502	6·4	45	0·6%	1·7%	14	3	5852	1·9	28	0·2%	1·8%	12	3
	Eastern Asia	807·4	126 874	10·9	81	1·1%	5·1%	6	3	54 547	4·1	62	0·5%	4·1%	8	2
	Southeastern Asia	328·3	62 456	17·2	131	1·9%	12·4%	2	2	35 738	10·0	144	1·2%	12·6%	2	2
	South-central Asia	954·1	120 924	13·0	97	1·4%	13·9%	2	2	75 133	8·2	119	0·9%	13·8%	2	2
	Western Asia	129·3	5092	4·1	31	0·4%	2·7%	12	5	2993	2·5	36	0·3%	3·3%	10	7
	Central-eastern Europe	154·6	35 940	16·0	114	1·6%	5·9%	5	2	16 011	6·1	83	0·7%	5·2%	8	1
	Northern Europe	53·1	6319	9·5	61	0·9%	2·1%	13	3	2060	2·1	32	0·2%	1·6%	17	2
	Southern Europe	78·3	9155	7·8	54	0·8%	2·3%	13	3	3512	2·2	33	0·2%	2·0%	15	2
	Western Europe	98·4	9658	6·8	48	0·7%	1·7%	15	4	4246	2·1	33	0·2%	1·8%	16	3
	Australia and New Zealand	14·8	1114	6·0	41	0·6%	1·5%	14	5	403	1·7	25	0·2%	1·6%	18	3
	Melanesia	5·2	1254	27·7	219	2·6%	15·4%	2	2	825	19·0	290	1·9%	17·5%	2	1
	Micronesia	0·3	51	18·6	141	2·1%	11·2%	3	2	22	7·8	114	1·0%	8·4%	3	4
	Polynesia	0·3	37	10·7	81	1·2%	5·0%	6	3	18	5·2	73	0·6%	4·9%	5	2

HDI=Human Development Index. ASIR=world age-standardised incidence rate. SIR=standardised incidence ratio. CIR=cumulative incidence rate of developing cervical cancer. ASMR=world age-standardised mortality rate. SMR=standardised mortality ratio. CMR=cumulative mortality rate of cervical cancer.

* Before age 75 years.

**Figure 1:**
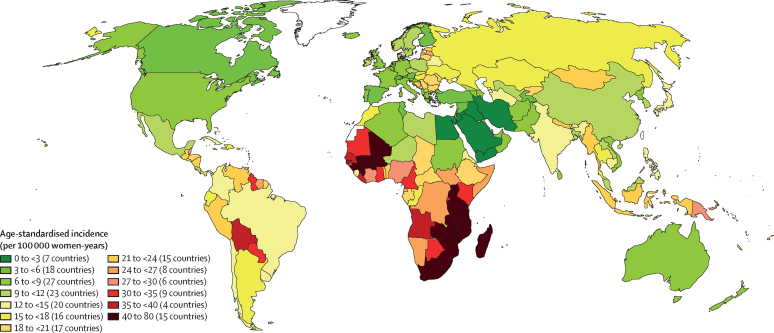
Geographical distribution of world age-standardised incidence of cervical cancer by country, estimated for 2018

**Figure 2:**
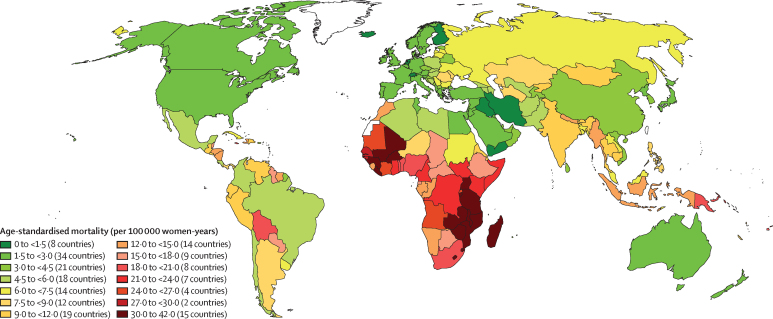
Geographical distribution of world age-standardised mortality rate of cervical cancer by country, estimated for 2018

The variations in rates are more striking when the focus is on subcontinents (Figure 1, Figure 2). Overall, the lowest incidence burden was observed in western Asia and the lowest mortality burden was observed in Australia–New Zealand (table). Rather modest incidences (ASIR <10 per 100 000) were also noted in Australia–New Zealand, northern America, western Europe, northern Africa, southern Europe, and northern Europe. The highest burden was observed in southern Africa and eastern Africa. A very high burden of the disease (ASIR ≥15 per 100 000) was also observed in western Africa, Melanesia, middle Africa, Micronesia, southeastern Asia, eastern Europe, the Caribbean, and South America (table).

**Figure 3:**
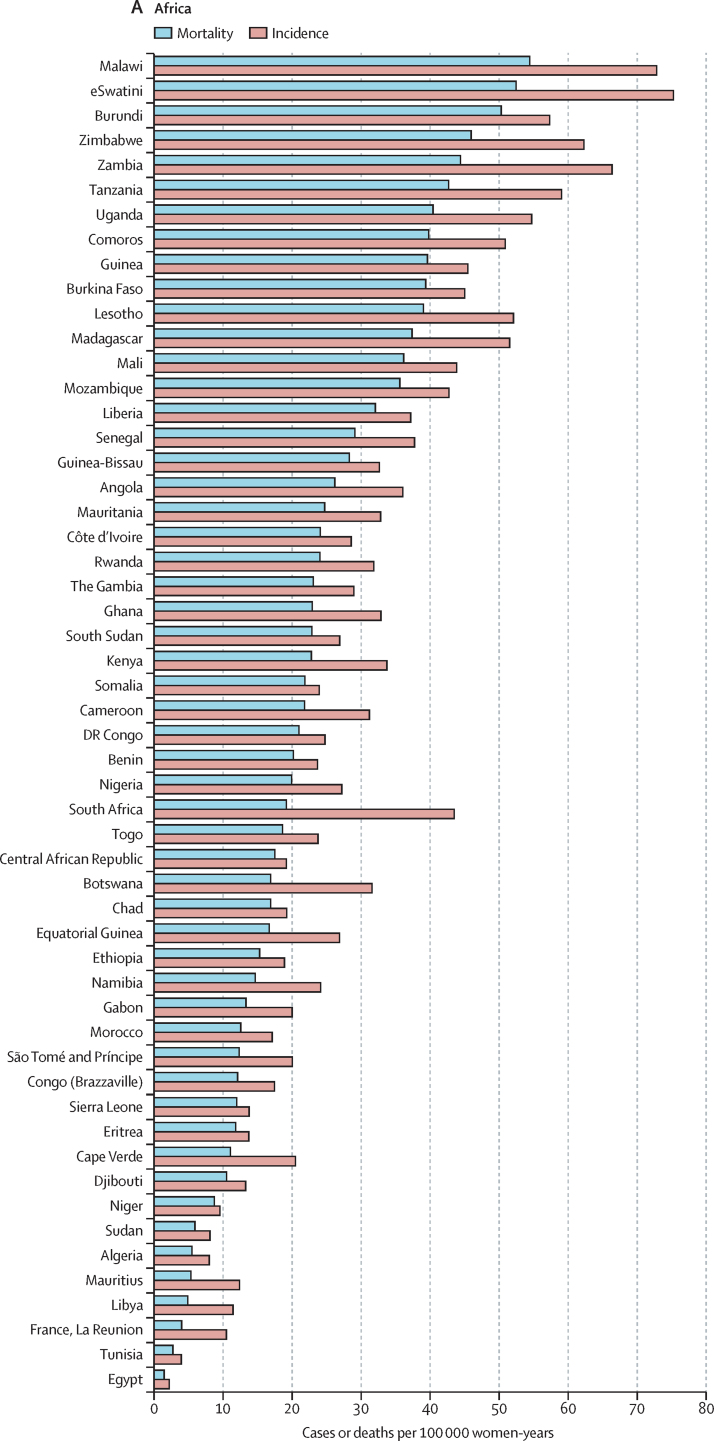

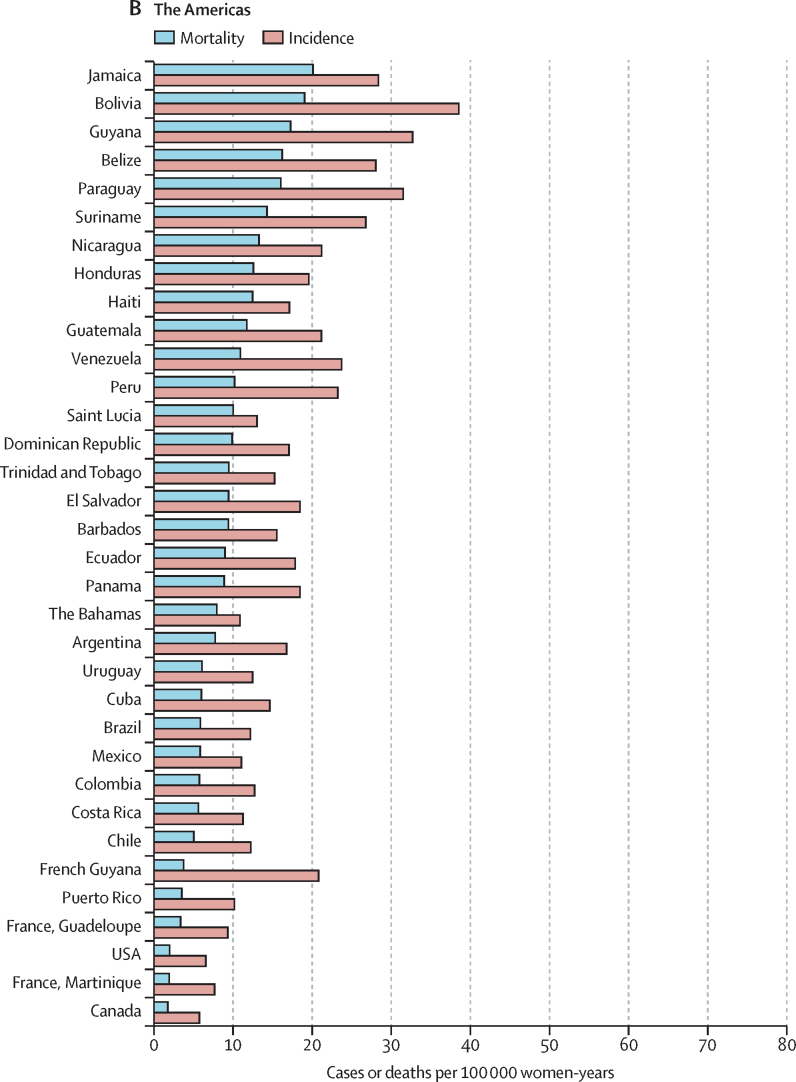

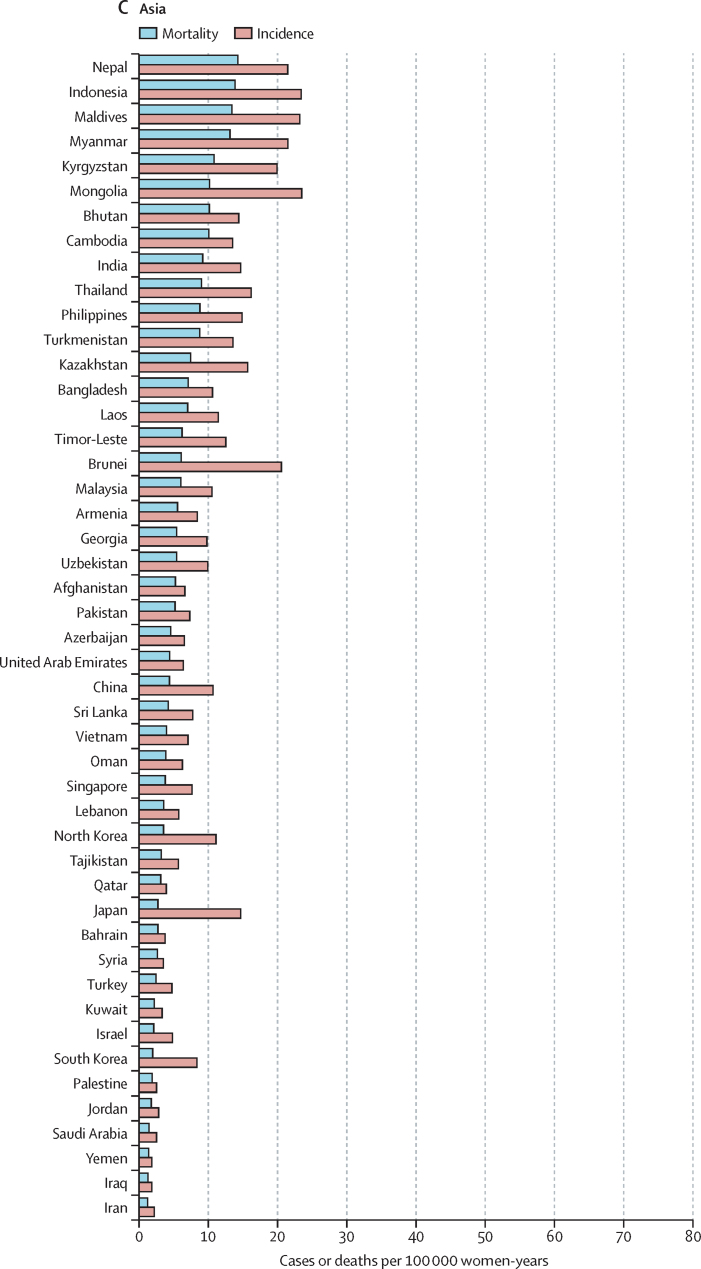

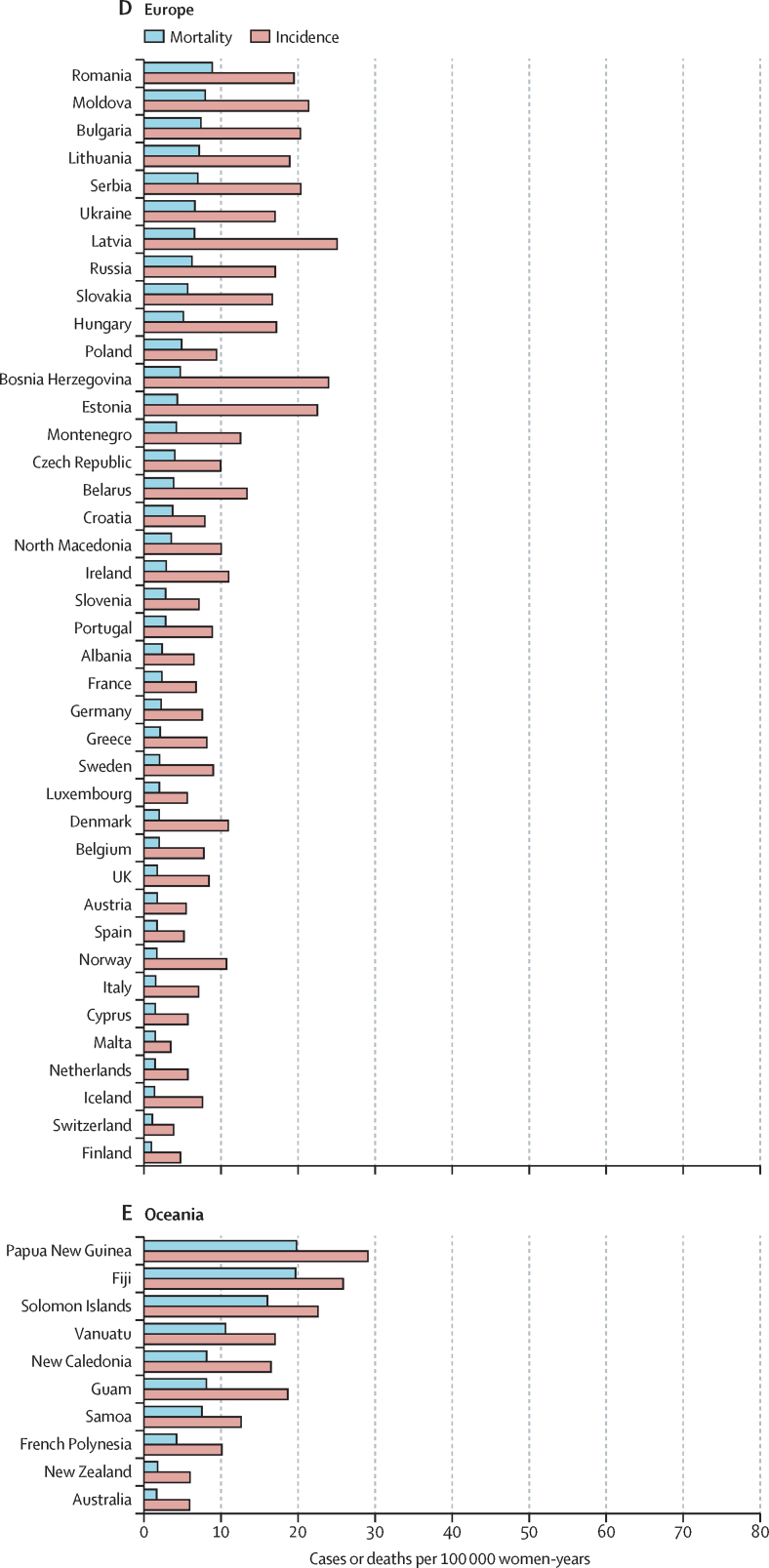
World age-standardised incidence and mortality rate for cervical cancer, estimates for 2018, ordered by country and ranked in descending order of mortality

The highest incidences (ASIR >40 per 100 000) were all found in countries from eastern, southern, or western Africa ((eSwatini, Malawi, Zambia, Zimbabwe, Tanzania, Burundi, Uganda, Lesotho, Madagascar, Comoros, Guinea, Burkina Faso, Mali, South Africa, and Mozambique; figure 3). China was the country with the highest number of cases (106 000), whereas India was the country with the highest estimated number of cervical cancer deaths (60 000; cervical cancer incidence and mortality statistics at country level are detailed in the appendix [pp 7–14]). China and India together contributed 35% to the global burden of cervical cancer cases and deaths.

The lowest ASIR values (<5 per 100 000 women) were estimated in 12 countries in western Asia or the western part of central-south Asia (Iraq, Yemen, Iran, Palestine, Saudi Arabia, Jordan, Kuwait, Syria, Bahrain, Qatar, Israel, and Turkey), two countries in north Africa (Egypt and Tunisia), one country in southern Europe (Malta), and one country in northern Europe (Finland). ASMR was significantly correlated with ASIR (p<0·0001) resulting in a very similar geographical distribution between the two (Figure 1, Figure 2). However, the range of variation was greater for SMR (ranging from 16 to 804) than for SIR (ranging from 13 to 570). The higher rates SMR values are in line with the observation that countries with higher ASIR had lower survival (approximated by the compliment of the ratio of mortality over incidence; p<0.0001).^25^

**Figure 4:**
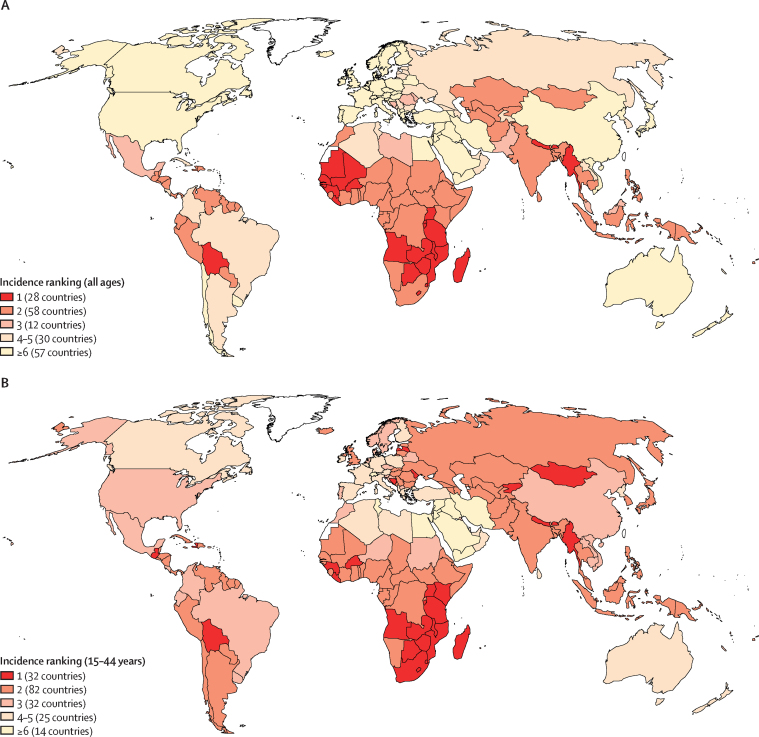
Ranking of cervical cancer incidence burden in 2018 relative to all other cancer sites in women of all ages (A) and aged 15–44 years (B)

Figure 4 displays the ranking of cervical cancer in each country among all cancer sites in women in terms of number of cases, for all ages, and for women aged 15–44 years. In 98 (52%) of 185 countries assessed, cervical cancer was among the three most frequent cancers in women of all ages (figure 4). However, in women younger than 45 years, cervix cancers ranked in the top three cancers in 146 countries (79%) worldwide.

**Figure 5:**
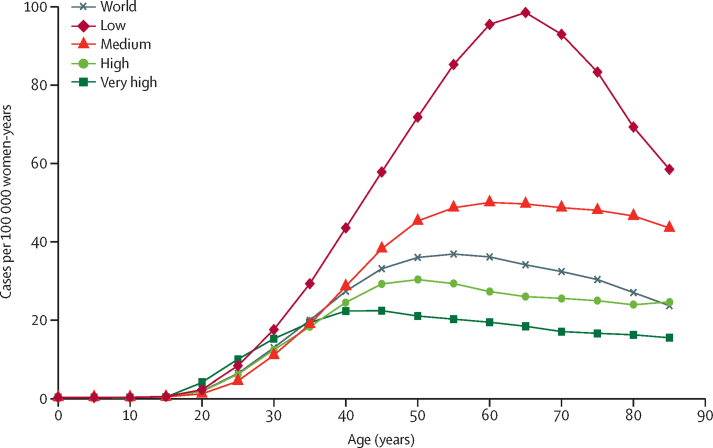
Age-specific incidence of cervical cancer worldwide and in terms of the four-tier HDI The four tiers of HDI are the following: very high (HDI ≥0·8), high (HDI <0·8 to ≥0·7), medium (HDI <0·7 to ≥0·55), and low (HDI <0·55). HDI=Human Development Index.

The age-specific incidence rate of cervix cancer starts rising after the age of 25 years (figure 5). In the highest-resource countries, a maximum of incidence is reached around the age of 40 years, whereas in lower-resource countries, rates continued to rise markedly up to ages 55–69 years. Globally, the average age at diagnosis of cervical cancer was 53 years, ranging from 44 years (Vanuatu) to 68 years (Singapore). The global average age at death from cervical cancer was 59 years, ranging from 45 years (Vanuatu) to 76 years (Martinique). The incidence peaked at ages 50–54 years at the global level. The country with the earliest peak was the UK (30–34 years), whereas a large group of countries had their maximal incidence in the age group of 85 years and older (data not shown).

## Discussion

With almost 0·6 million cases and 0·3 million deaths per year, cervical cancer continues to constitute a major public health problem, ranking as the fourth most common cause of cancer incidence and mortality in women worldwide. Notwithstanding the caveats of interpreting estimates from different years given the variations in source information, the absolute number of cases of cervix uteri cancer worldwide estimated in GLOBOCAN increased over time (471 000 in 2000, 529 000 in 2008, and 570 000 in 2018).^1^, ^26^ This rise could be driven by the growth and aging of the global population,^24^ and cervical cancer incidences have tended to decrease (ASIR 16·2 per 100 000 women in 2002,^28^ 15·2 in 2008,^1^ and 13·1 in 2018). In the previous estimates for 2008, cervical cancer still ranked as the third most frequent malignant tumour, accounting for 8·7% of all cancers in women (excluding non-melanoma skin cancers), but dropped to fourth ranking in 2018, with 6·9% of the total cancer burden. Declining incidences were observed in most world regions in the first decade of the 21st century, but exceptions were also seen in eastern Europe and sub-Saharan Africa.^29^ The proportion of cervical cancer deaths among all cancer deaths decreased from 8·2% in 2008, to 7·5% in 2018, although the fourth place ranking of cervical cancer deaths was retained.

Because the mean age at diagnosis of cervical cancer is quite low compared with that of most other major cancer types, it generates proportionally greater loss of life-years.^30^ Age-specific analyses (figure 5) clearly indicated that cervical cancer occurred across a range of ages during which adult women have many economic and caregiving responsibilities for their families. The absence of a further rise in incidence after age 40 years in high-resource countries could reflect cancers prevented by screening, although hysterectomy might have also partly contributed to a reduced number of cervical cancer cases.

Considerable rate variations were noted, with incidences ranging from less than 3 to more than 70 per 100 000 women. Mortality from cervical cancer is the malignancy with the largest inter-country range of variation among all cancers.^27^ Cervical cancer remains the leading cause of cancer death in women in 42 lower-resource countries (appendix p 3), by contrast with being the 19th most common cause in Finland (a high-resource country). Such remarkable geographical contrasts reflect differences in exposure to risk factors and serious inequalities in access to adequate screening and effective cancer treatment facilities.^31^, ^32^ Sexually transmitted infection with high-risk HPV types is the main aetiological factor for cervical cancer.^33^, ^34^ We plotted the prevalence of high-risk HPV by subcontinent derived from a meta-analysis updated in 2018, involving 2·4 million women with normal cervical cytology against the respective standardised cervical incidence.^1^, ^35^ The scatter plot (appendix p 4) showed a clear positive correlation (r=0·70).

Other cofactors, such as some sexually transmittable infections (HIV and *Chlamydia trachomatis*), smoking, and oral hormonal contraception, might also contribute to changes and contrasts in the global cervical cancer burden.^36^, ^37^, ^38^ However, other putative factors related to socioeconomic development and transitions to a lifestyle more typical of high-income countries (including reproductive and sexual factors) seem to underpin major changes in cancer risk, the effect of which was seen in the lowering of cervical cancer rates over time and concomitant rises in breast cancer rates in several countries with emerging economies.^39^, ^40^ These societal changes are clearly illustrated by the cervical cancer incidence trends in India, which have declined in urban areas but have stayed stable in rural areas.^41^ A notable and novel public health concern is the observation of an upward trend in cervical cancer incidence in several countries with established preventive programmes, which might be explained by increased exposure to HPV insufficiently compensated by cytological screening.^42^, ^43^, ^44^

Parts of western Asia and northern Africa with incidence of cervical cancer have a low prevalence of HPV (figure 1), most plausibly explained by societal factors related to sexual behaviour.^45^ These countries also exhibit low rates of other sexually related infections, such as HIV.^46^ By contrast, in areas in sub-Saharan Africa, Latin America, and south Asia, the high cervical cancer rates probably reflect an elevated background risk, explained by high rates of HPV and HIV transmission.^47^

The low rates of cervical cancer in north America, northern and western Europe, and Australia and New Zealand are probably the result of successful cytological screening.^18^, ^48^, ^49^, ^50^, ^51^ These screening programmes have counteracted increased exposure to risk factors among generations born after 1945, as established from age-period-cohort analyses^50^, ^52^, ^53^ and from HPV prevalence surveys in archived biospecimens.^54^ However, where screening, management of patients with positive screen results, or both were of poor quality, the cohort effect was not balanced in the same way, resulting in trends that were slightly declining, stable, or even increasing, as observed in Ireland, Portugal, and in several Baltic and eastern European countries, where the burden of cervical cancer is among the highest on the European continent.^55^, ^56^, ^57^, ^58^

Although the number of certified cancer registries and the quality of registered incidence and mortality data have improved over time, only 24% of countries provided directly usable national incidence data and 44% did so for mortality data (appendix pp 5–6). No information could be identified for incidence in 32 countries and for mortality in 84 countries, thus estimates have been computed either from modelling or from neighbouring countries. The GLOBOCAN 2018 estimations can be considered the best possible given the data available; however, they should be interpreted with caution, because their reliability is determined by the quality and completeness of registration and by the appropriateness of external data in the absence of recorded data.^17^

A key concern in comparative assessments of cervical cancer mortality is the accuracy of cause of death certification, because a large proportion of deaths are assigned to uterine cancer without specification of exact topographic origin. Countries with a very high burden of cervical cancer often correspond to areas where local data are either absent or of suboptimal quality (appendix pp 5–6). Local difference in the practice of hysterectomy might have some effect on cervical cancer incidence reported in our study, but could not be accounted for.^59^ Finally, we note that the successive iterations of GLOBOCAN present contemporary estimate of the global burden of cancer by use of the best available sources; however, they are not a good basis for time trend analyses. To assess temporal aspects and, particularly, the effects of interventions, the use of long-term time series from high-quality registries is recommended, such as those compiled in successive editions of Cancer Incidence in Five Continents.

Accumulated evidence indicating that screening with HPV tests is more effective in preventing future cervical precancerous lesions and invasive cancers than screening with Pap smears^9^, ^10^ has been translated into new national and international recommendations to use validated HPV assays as the preferred test for primary screening.^11^, ^12^, ^60^ Moreover, HPV testing can be done on specimens taken by the woman herself, offering opportunities—in both resource-rich and poor countries—to reach women who otherwise would not participate in screening by enabling self-sampling.^61^ Additionally, systematic reviews of randomised trials completed with observational data from vaccination programmes have shown the protective effect of HPV vaccines against HPV infection and associated precancers, particularly among girls and young women not yet infected with HPV vaccine types.^3^, ^62^ Although some indicative observations of a reduced incidence of cervical cancer in vaccinated populations exist,^63^, ^64^ it is still too early to observe a clear vaccination effect on the existing HPV-related cancer incidences.

The availability of these new powerful tools for primary and secondary prevention and the enduring large burden of cervical cancer worldwide have motivated WHO to initiate an ambitious plan to eliminate cervical cancer as a public health problem in the 21st century by reducing the global annual age-standardised incidence to 4 per 100 000 women.^65^ By vaccinating 90% of all girls by the age of 15 years, screening 70% of women twice in a life time (at ages 35 years and 45 years) with a precision test (ie, a validated HPV assay), and treating 90% of precancerous cervical lesions detected during screening, this WHO goal might become possible to reach.^66^ In several higher-resource countries, including the USA, Australia, New Zealand, Turkey, and several western European nations, HPV-based screening, combined with or without cytology, are being implemented, with several countries including options to offer self-sampling kits.^61^ In 2018, only a quarter of 10-year-old girls globally live in the 85 countries that have introduced HPV vaccination. This proportion varies between 13% in low-resource countries to 82% in high-resource countries.^67^, ^68^ Mathematical modelling, using previous GLOBOCAN estimates, predicts that the WHO threshold of 4 per 100 000 women-years could be reached in very high HDI areas by 2055–59, whereas in low HDI countries, the elimination goal could be reached closer to the end of this century.^66^

Several new assays allowing point-of-care HPV testing or visual devices with automated interpretation of cervical images that are accurate, robust, user-friendly, and affordable (which are being developed) need urgent validation and should be manufactured at large scale if evaluations are successful.^69^, ^70^, ^71^ Additional implementation research and actions are needed to inform on best evidence-based practices that fit various situations. These areas include how to best integrate policies of vaccination and screening, including the screening of high-risk populations with elevated HIV prevalence; the risk-based management of women positive for high-risk HPV by use of appropriate triage procedures or through screen-and-treat approaches; and adapting screening policies in settings where cervical cancer risk is low because of successful prevention strategies. Other key issues warranting further study are assessing the effect of self-sampling kits in yielding improved participation in the target populations; strategies ensuring high compliance, with treatment of precancer lesions with safe and efficacious procedures;^60^, ^72^ and assessing the level of access to treatment and palliative care centres among patients with invasive tumours.^73^, ^74^, ^75^

Cervical cancer kills approximately 300 000 women and affects nearly 600 000 women yearly, particularly middle-aged women and those living in lower-resource settings. However, most cervical cancers and related deaths can be avoided by integrated HPV-based screening and vaccination. WHO is developing a global plan of action to engage stakeholders and mobilise resources to make cervical cancer a rare disease globally through an ambitious scale-up of national services over the next decades. The GLOBOCAN 2018 figures presented in this study are pivotal to provide a baseline for the targets of the global strategy that will be submitted for ratification by WHO Member States at the 2020 World Health Assembly.
