# Correction to *Lancet Glob Health* 2020; 8: e191–203

**DOI:** 10.1016/S2214-109X(21)00554-4

**Published:** 2021-12-14

*Arbyn M, Weiderpass E, Bruni L, et al. Estimates of incidence and mortality of cervical cancer in 2018: a worldwide analysis.* Lancet Glob Health *2020; **8:** e191–203*—In this Article, in the Acknowledgments section, support from the RISCC Network (grant 847845) was incorrectly omitted. This correction has been made as of Dec 14, 2021.

