# Supplementary material for: Estimates of incidence and mortality of cervical cancer in 2018: a worldwide analysis
Source: Lancet Glob Health. 2019 Dec 4;8(2):e191–203. doi: 10.1016/S2214-109X(19)30482-6 (PMC7025157; doi:10.1016/S2214-109X(19)30482-6)
Supplement: Supplementary appendix [file mmc1.pdf]

THE LANCET
Global Health

Supplementary appendix

This appendix formed part of the original submission and has been peer reviewed. We post it as supplied by the authors.

Supplement to: Arbyn M, Weiderpass E, Bruni L, et al. Estimates of incidence and mortality of cervical cancer in 2018: a worldwide analysis. *Lancet Glob Health* 2019; published online Dec 4. http://dx.doi.org/10.1016/S2214-109X(19)30482-6.

Supplementary appendix

Worldwide burden of cervical cancer in 2018

Marc Arbyn[1*], Elisabete Weiderpass[2], Laia Bruni[3,4], Silvia de Sanjosé[4,5], Mona Saraiya[6], Jacques Ferlay[2], Freddie Bray[2]

1 Unit of Cancer Epidemiology, Belgian Cancer Centre, Sciensano, Brussels, Belgium;
2 International Agency for Research on Cancer, Lyon, France;
3 Consortium for Biomedical Research in Epidemiology and Public Health (CIBERESP), Spain;
4 Unit of Infections and Cancer, Catalonian Institute of Oncology, Barcelona, Spain;
5 PATH, Seattle, USA;
6 Centers for Disease Control and Prevention, Division of Cancer Prevention and Control, Atlanta, USA.

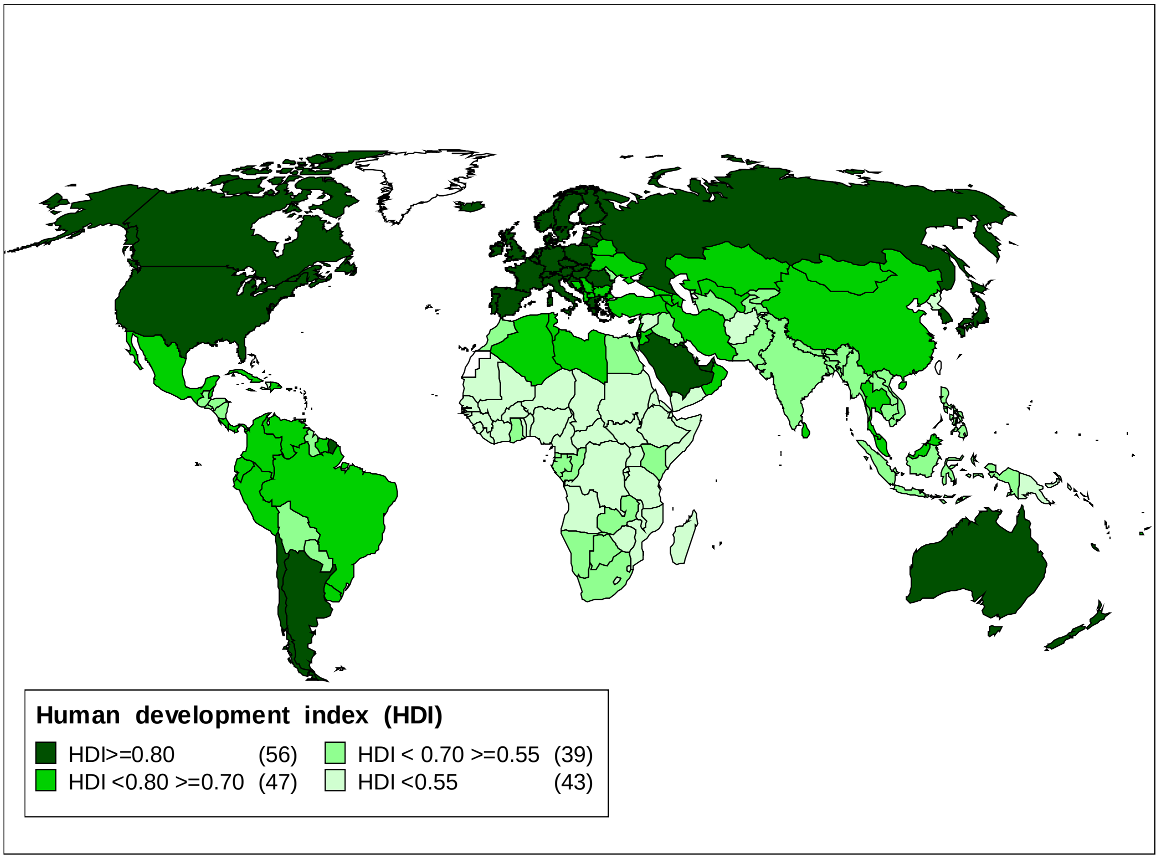

Supplementary Figure 1. Human development index (HDI) attributed to the 185 countries included in the study of the burden of cervical cancer. Sources: United Nations Development Programme, New York, 2016 and International Agency for Research on Cancer, Lyon, 2018 (Bray F, et al, CA Cancer J Clin 2018).

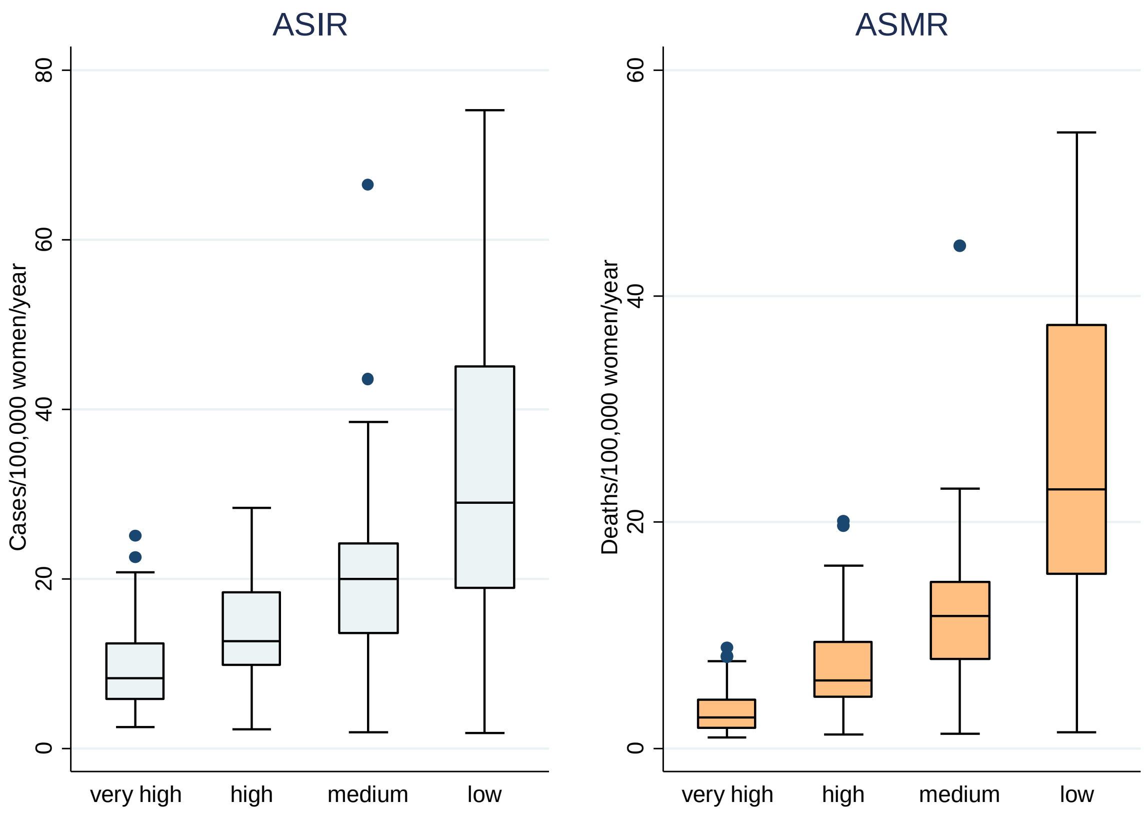

Supplementary Figure 2. Distribution of the world age-standardised incidence (ASIR, left) and mortality rate (ASMR, right) by category of socio-economic development (expressed by the human development index).

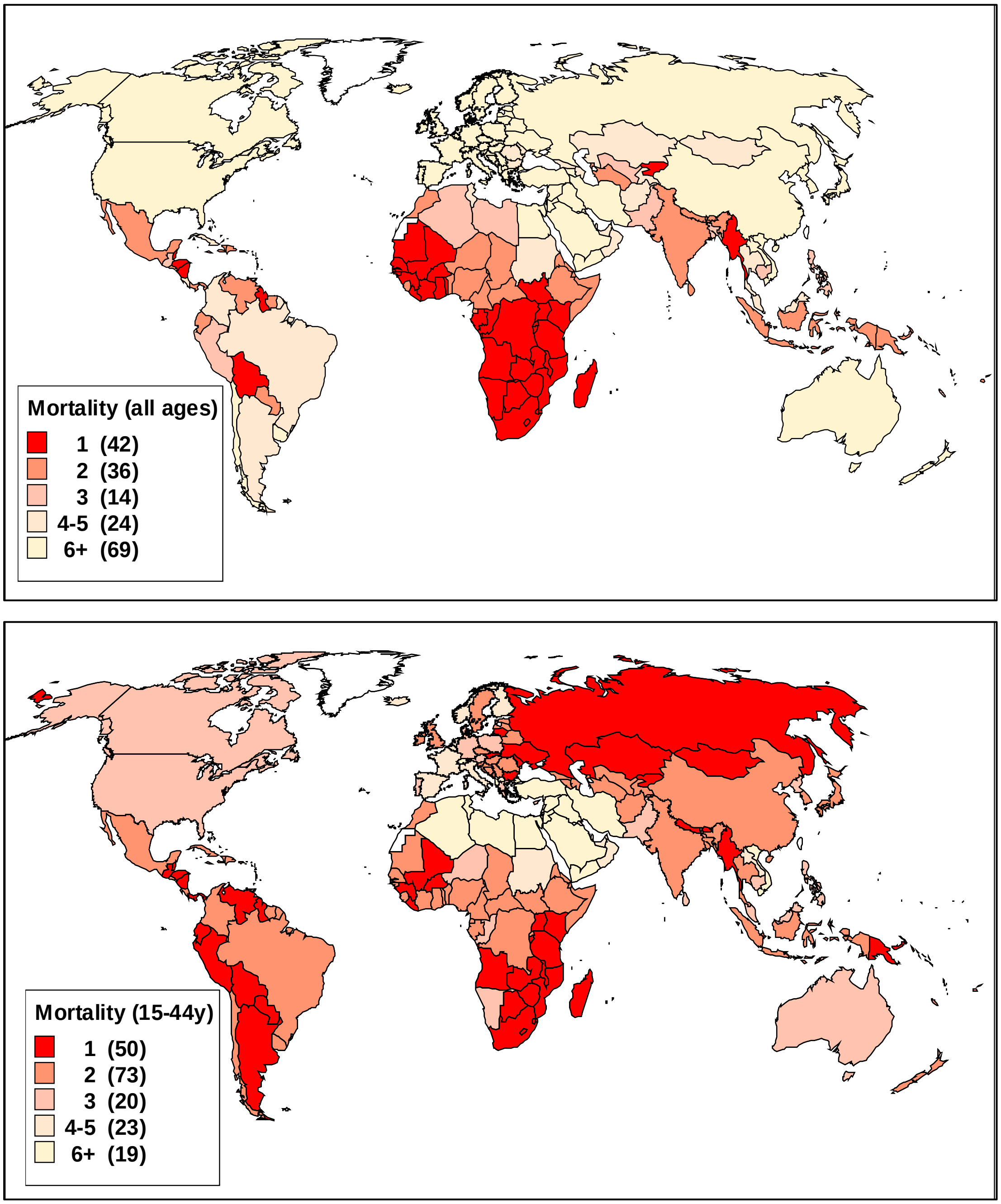

Supplementary Figure 3. Ranking of the cervical cancer mortality burden in 2018 relative to all other cancer deaths in women of all ages (top) and aged 15-44 years (bottom).

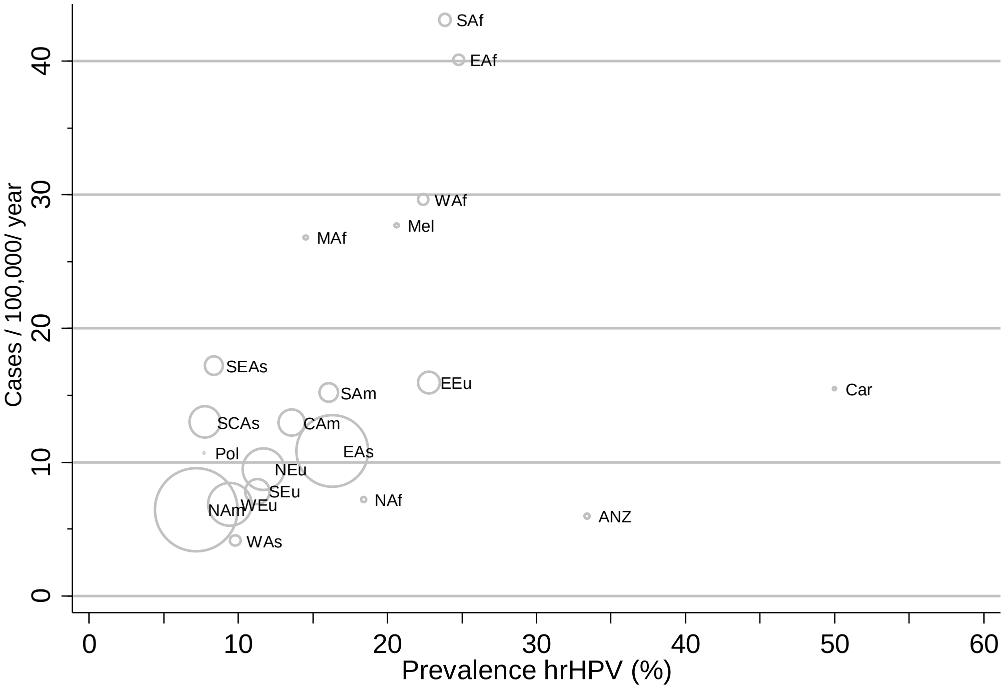

Supplementary Figure 4. Age-standardised incidence (computed using the world reference population) of cervical cancer as a function of the prevalence of high-risk HPV infection in women with a normal Pap smear, by sub-continent.

The scatter points are scaled according to the number of tested women per subcontinent, included in the meta-analysis (Bruni, Lancet Global Health et al, 2016; 4: e453-e463; update presented at the 32nd International Papillomavirus Conference, Sydney, 2-6 October, 2018).

Note the excessively high pooled hrHPV prevalence in the Carribean and Australia/New-Zealand because of inclusion of specific populations. However, because of the small study sizes from these two subcontinents, the weighted regression was hardly influenced.

EAf=Eastern Africa; NAf=Northern Africa; SAf=Southern Africa; CAm=Central America; Car=Carribean; NAm=Northern America; SAm=Southern America; EAs=Eastern Asia; SCAs: South-Central Asia; SEAs=South-Eastern Asia; WAs= Western Asia; EEu= Eastern Europe; NEu= Northern Europe; SEu: Southern Europe; WEu= Western Europe).
Prevalence of hrHPV estimates derived from Bruni et al (IPV 2018, Sydney)[32].

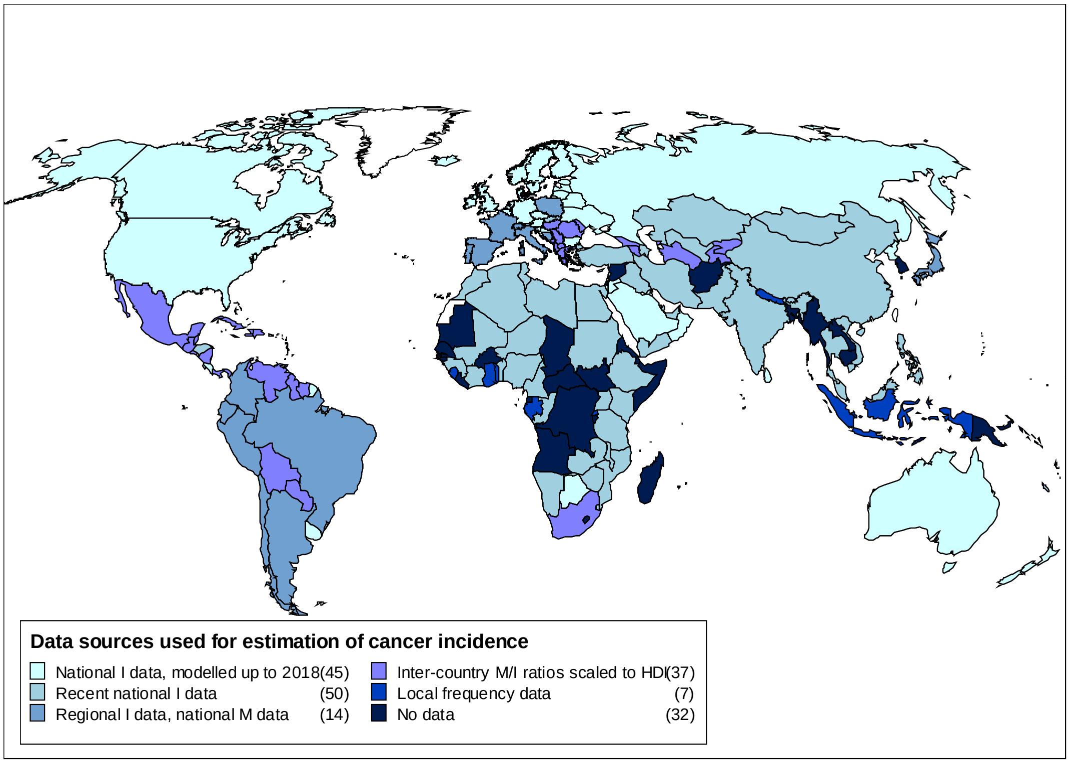

Supplementary Figure 5. Availability of data per country to estimate cervical cancer incidence.

HDI: human development index; I: incidence; M: mortality.

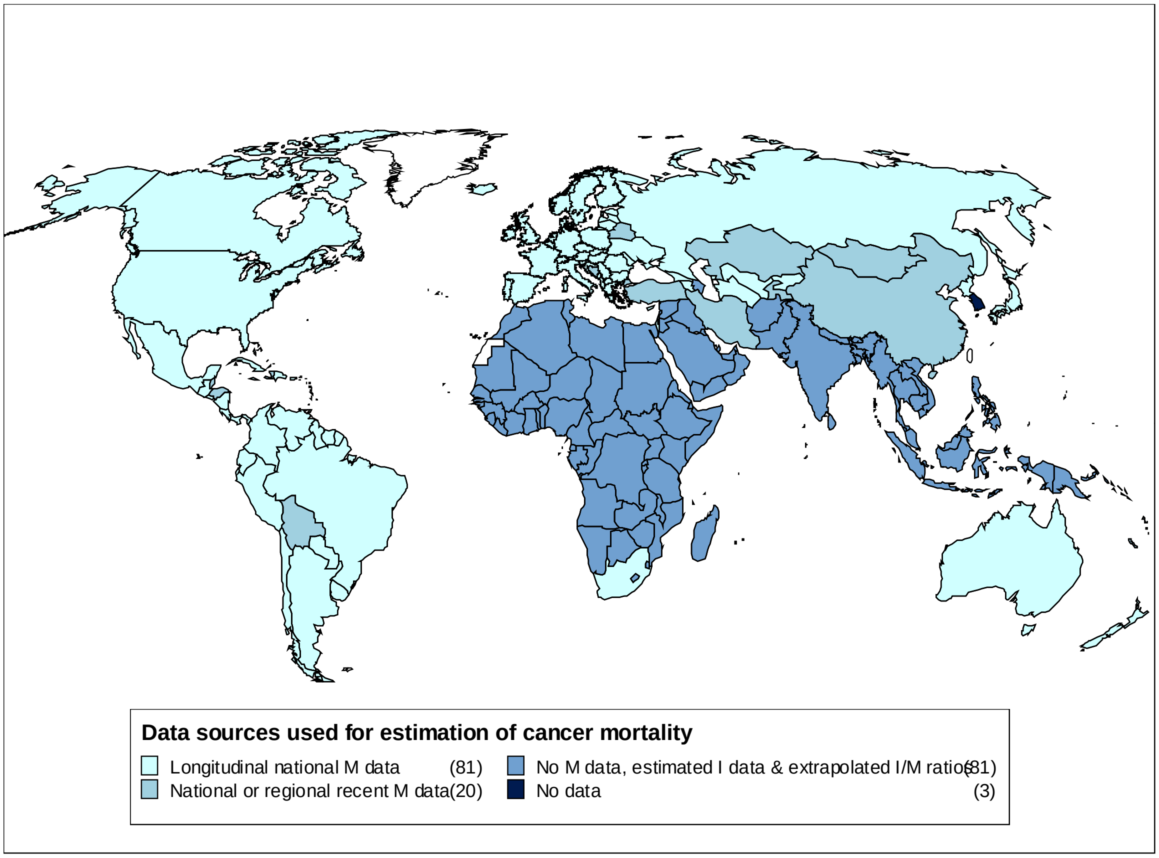

Supplementary Figure 6. Availability of data per country to estimate cervical cancer mortality.
I: incidence; M: mortality.

Supplementary Table 1. Burden of cervical cancer incidence and mortality in 2018, by country, ordered by subcontinent: female population size, number of cases of and deaths from cervical cancer, world-age-standardised incidence (ASIR) and mortality (ASMR) rates; standardised incidence and mortality ratios (SIR; SMR); cumulative rate of developing cervical cancer (CIR) or dying from cervical cancer (CMR) before the age 75 years; percentage and rank that cervical cancer takes among all cancers cases or cancer deaths[†].

Country	Total female population*	Nb of cases	ASIR	SIR	CIR (%)	% of all cancers	Rank (all ages)	Rank (15-44y)	Nb of deaths	ASMR	SMR	CIR (%)	% of all cancers	Rank (all ages)	Rank (15-44y)
**Eastern Africa**															
Burundi	5.7	1,859	57.4	416	6.4	38	1	1	1,528	50.3	724	5.8	40.5	1	1
Comoros	0.4	141	50.9	370	5.6	46	1	1	103	39.8	567	4.6	46.6	1	1
Djibouti	0.5	52	13.3	95	1.5	12	2	2	39	10.6	147	1.3	13.7	2	2
Eritrea	2.6	218	13.8	97	1.6	11	2	2	178	11.9	165	1.4	12.3	2	2
Ethiopia	53.8	6,294	18.9	135	2.1	14	2	2	4,884	15.3	216	1.8	15.8	2	2
France, La Reunion	0.5	70	10.5	88	1.1	6	4	2	33	4	73	0.4	6.2	6	4
Kenya	25.6	5,250	33.8	233	3.8	19	2	1	3,286	22.8	314	2.7	17.7	1	1
Madagascar	13.2	4,353	51.6	374	5.7	41	1	1	2,940	37.4	528	4.3	40.9	1	1
Malawi	9.7	4,163	72.9	553	7.9	35	1	1	2,879	54.5	804	6.2	36.1	1	1
Mauritius	0.6	120	12.4	104	1.4	8	3	2	56	5.4	85	0.7	8	3	2
Mozambique	15.6	4,291	42.8	330	4.4	31	1	1	3,376	35.7	534	3.8	34.2	1	1
Rwanda	6.4	1,304	31.9	223	3.7	21	1	2	921	24.1	333	2.9	21.5	1	2
Somalia	7.6	989	24.0	172	2.7	16	2	2	875	21.9	320	2.5	17.3	2	2
South Sudan	6.4	1,101	26.9	196	3.0	20	2	2	888	22.9	327	2.7	21.8	1	2
Tanzania	29.9	9,772	59.1	404	6.9	40	1	1	6,695	42.7	577	5.2	41.1	1	1
Uganda	22.2	6,413	54.8	413	5.8	36	1	1	4,301	40.5	601	4.5	36.6	1	1
Zambia	8.9	2,994	66.4	442	7.7	42	1	1	1,839	44.5	585	5.3	43.6	1	1
Zimbabwe	8.7	3,186	62.3	431	7.0	29	1	1	2,151	46	628	5.4	29.5	1	1
**Middle Africa**															
Angola	15.7	2,949	36.1	244	4.1	33	1	1	1,987	26.2	350	3.1	34.1	1	1
Cameroon	12.3	2,356	31.3	230	3.4	26	2	2	1,546	21.9	317	2.5	25.9	2	2
Central African Rep.	2.4	276	19.2	139	2.2	18	2	2	246	17.5	249	2	20.3	2	2

Country	Total female population*	Nb of cases	ASIR	SIR	CIR (%)	% of all cancers	Rank (all ages)	Rank (15-44y)	Nb of deaths	ASMR	SMR	CIR (%)	% of all cancers	Rank (all ages)	Rank (15-44y)
Chad	7.7	742	19.3	135	2.2	17	2	2	618	16.9	239	2	18.7	2	2
Congo, DR	42.1	5,762	24.8	172	2.9	22	2	2	4,665	21.1	288	2.6	23.7	1	2
Congo, Republic of	2.7	278	17.5	113	2.1	22	2	2	187	12.1	157	1.5	24.2	1	3
Equatorial Guinea	0.6	100	26.9	201	2.9	24	2	2	57	16.7	246	1.9	21.3	2	2
Gabon	1	156	20.0	151	2.1	18	2	2	100	13.4	197	1.5	18.7	1	2
**Northern Africa**															
Algeria	20.8	1,594	8.1	58	0.9	6	4	5	1,066	5.5	76	0.7	8.1	3	8
Egypt	49.1	969	2.3	16	0.2	2	14	11	631	1.5	21	0.2	1.6	12	12
Libya	3.2	319	11.5	75	1.3	10	3	5	127	4.9	63	0.6	8.6	3	10
Morocco	18.3	3,388	17.2	130	2.0	12	2	3	2,465	12.6	180	1.5	17.5	2	2
Sudan	20.8	1,084	8.2	57	1.0	7	2	3	745	6	80	0.8	7.7	4	5
Tunisia	5.9	285	4.0	31	0.5	4	4	9	199	2.8	40	0.3	5	5	12
**Southern Africa**															
Botswana	1.2	333	31.6	242	3.3	32	1	1	166	16.9	248	1.8	32.1	1	1
Eswatini‡	0.7	380	75.3	570	7.0	55	1	1	238	52.5	751	4.9	56.8	1	1
Lesotho	1.2	477	52.1	410	5.0	43	1	1	346	39.1	591	3.9	45.2	1	1
Namibia	1.3	236	24.2	177	2.6	22	2	2	135	14.7	208	1.6	23.2	1	3
South Africa	29.2	12,983	43.5	341	4.3	23	2	1	5,595	19.2	286	2	19.3	1	1
**Western Africa**															
Benin	5.8	783	23.7	156	2.8	19	2	2	652	20.2	266	2.4	22.5	2	2
Burkina Faso	9.9	2,517	45.1	316	5.1	34	1	1	2,081	39.4	552	4.7	36.4	1	1
Cape Verde	0.3	50	20.5	156	2.3	17	1	1	26	11.1	160	1.4	12	1	1
Cote d'Ivoire	12.3	1,789	28.6	178	3.5	22	2	2	1,448	24.1	309	3	25.4	1	2
Ghana	14.8	3,151	32.9	214	4.1	23	2	2	2,119	23	301	2.9	25.5	1	2
Guinea	6.5	1,810	45.5	320	5.1	45	1	1	1,509	39.7	553	4.6	47	1	1
Guinea-Bissau	1	191	32.7	224	3.8	30	1	2	157	28.3	387	3.4	32.9	1	2
Liberia	2.4	548	37.2	254	4.3	33	1	1	449	32.1	435	3.9	35.2	1	1
Mali	9.5	2,206	43.9	307	5.1	27	1	2	1,704	36.2	503	4.4	29.3	1	1

Country	Total female population*	Nb of cases	ASIR	SIR	CIR (%)	% of all cancers	Rank (all ages)	Rank (15-44y)	Nb of deaths	ASMR	SMR	CIR (%)	% of all cancers	Rank (all ages)	Rank (15-44y)
Mauritania	2.3	481	32.9	226	3.7	30	1	2	341	24.8	334	2.9	31.3	1	2
Niger	11.1	543	9.6	69	1.1	11	2	3	476	8.8	126	1.1	12.5	2	3
Nigeria	96.6	14,943	27.2	183	3.3	21	2	2	10,403	20	270	2.5	25.1	2	2
Sao Tome & Principe	0.1	16	20.1	179	1.4	25	1	1	9	12.4	211	0.8	23.1	1	1
Senegal	8.3	1,876	37.8	257	4.4	28	1	2	1,367	29.1	392	3.6	30.2	1	2
Sierra Leone	3.9	299	13.8	91	1.6	12	2	2	242	12	160	1.4	14.1	2	2
The Gambia	1.1	184	29.0	217	2.9	54	1	1	132	23.1	341	2.4	51.8	1	1
Togo	4	568	23.8	159	2.7	21	2	2	414	18.7	247	2.2	23	1	2
**Carribean**															
Bahamas	0.2	29	10.9	84	1.1	7	4	2	23	7.9	122	0.9	9.9	2	5
Barbados	0.1	38	15.5	132	1.5	7	4	4	27	9.4	157	0.9	7.6	3	5
Cuba	5.7	1,231	14.6	106	1.4	6	4	2	597	6	88	0.7	5.5	4	2
Dominican Republic	5.5	981	17.1	136	1.7	11	2	2	571	9.9	150	1.1	11	2	2
France, Guadeloupe	0.2	39	9.3	80	1.0	5	5	3	19	3.3	64	0.3	5.2	6	5
France, Martinique	0.2	32	7.6	73	0.7	4	10	3	14	1.9	52	0.1	3.5	10	28
Haiti	5.6	835	17.1	133	1.8	13	2	1	563	12.5	179	1.4	12.1	2	2
Jamaica	1.5	486	28.4	220	2.9	13	3	2	361	20.1	296	2.3	16.3	2	2
Puerto Rico	1.9	262	10.2	74	0.9	4	7	3	114	3.5	54	0.4	4.6	7	2
Saint Lucia	0.1	15	13.0	98	1.3	9	3	2	12	10	143	1.3	13.5	2	2
Trinidad and Tobago	0.7	140	15.2	115	1.5	8	4	2	97	9.4	144	1	10.1	2	2
**Central America**															
Belize	0.2	46	28.0	211	2.5	26	2	2	25	16.2	239	1.4	26.3	1	1
Costa Rica	2.5	351	11.2	88	1.1	6	5	3	192	5.6	88	0.6	7.4	4	2
El Salvador	3.4	724	18.5	153	1.8	12	2	2	386	9.4	151	0.9	10.8	2	2
Guatemala	8.8	1,503	21.1	163	2.2	17	2	1	793	11.7	172	1.3	16.1	3	1
Honduras	4.7	804	19.6	151	2.0	15	2	2	480	12.5	182	1.4	15.8	1	1
Mexico	65.7	7,869	11.0	86	1.1	8	3	3	4,121	5.8	86	0.6	9.7	2	2
Nicaragua	3.2	677	21.2	165	2.2	16	2	2	409	13.3	196	1.5	16.9	1	1
Panama	2.1	432	18.4	145	1.8	11	2	2	213	8.8	134	0.9	11.9	2	1

Country	Total female population*	Nb of cases	ASIR	SIR	CIR (%)	% of all cancers	Rank (all ages)	Rank (15-44y)	Nb of deaths	ASMR	SMR	CIR (%)	% of all cancers	Rank (all ages)	Rank (15-44y)
**South America**															
Argentina	22.8	4,484	16.7	126	1.7	7	4	2	2,231	7.7	109	0.8	6.7	5	1
Bolivia	5.6	1,949	38.5	291	4.0	23	1	1	1,022	19	291	2	19.6	1	1
Brazil	107.3	16,298	12.2	94	1.3	6	4	3	8,079	5.8	86	0.6	7.1	4	2
Chile	9.2	1,549	12.2	98	1.2	6	6	2	725	5	80	0.5	5.4	7	2
Colombia	25.1	3,853	12.7	98	1.3	7	4	3	1,775	5.7	85	0.6	7.7	5	2
Ecuador	8.4	1,612	17.8	142	1.9	11	2	3	838	9	139	0.9	11.4	2	1
French Guyana	0.1	29	20.8	157	2.2	11	2	2	5	3.7	53	0.4	7.4	5	2
Guyana	0.4	124	32.7	245	3.7	26	2	2	64	17.3	246	2	21.5	1	1
Paraguay	3.4	1,033	31.5	251	3.1	18	2	2	519	16	243	1.7	19.6	2	1
Peru	16.3	4,103	23.2	181	2.4	12	2	2	1,836	10.2	153	1.1	10.9	3	1
Suriname	0.3	85	26.8	204	2.9	17	2	2	47	14.3	212	1.7	16.5	2	2
Uruguay	1.8	288	12.4	92	1.2	4	6	3	168	6	88	0.6	4.4	7	2
Venezuela	16.3	4,174	23.7	184	2.4	13	2	2	1,926	10.9	161	1.2	12.9	2	1
**North America**															
Canada	18.6	1,434	5.7	39	0.5	1	14	4	586	1.7	26	0.2	1.5	16	3
USA	165	14,065	6.5	46	0.6	2	14	3	5,266	1.9	29	0.2	1.8	12	3
**Eastern Asia**															
China	690.2	106,430	10.7	81	1.1	6	6	3	47,739	4.4	66	0.5	4.5	8	2
Japan	65.1	13,277	14.7	92	1.4	4	9	2	4,088	2.7	43	0.3	2.4	11	2
Korea, DPR of	13.1	1,922	11.1	84	1.1	7	4	2	676	3.6	53	0.4	3.9	8	2
Korea, Republic of	25.6	3,348	8.4	63	0.8	3	10	4	1,029	2	33	0.2	3.1	10	3
Mongolia	1.6	370	23.5	175	2.5	14	2	1	150	10.2	145	1.1	9	4	1
**South-Eastern Asia**															
Brunei Darussalam	0.2	52	20.6	165	2.1	10	3	2	14	6.1	90	0.8	7.8	4	1
Cambodia	8.3	993	13.5	99	1.5	12	2	3	708	10.1	140	1.2	12.4	3	3
Indonesia	132.5	32,469	23.4	175	2.6	18	2	2	18,279	13.9	194	1.7	18.7	2	2

Country	Total female population*	Nb of cases	ASIR	SIR	CIR (%)	% of all cancers	Rank (all ages)	Rank (15-44y)	Nb of deaths	ASMR	SMR	CIR (%)	% of all cancers	Rank (all ages)	Rank (15-44y)
Lao PDR	3.5	320	11.4	83	1.3	8	4	3	182	7	97	0.8	7.4	6	4
Malaysia	15.5	1,682	10.5	79	1.2	7	3	2	944	6	86	0.7	7.6	4	3
Myanmar	27.6	6,472	21.5	166	2.2	19	1	1	3,856	13.1	192	1.5	16.3	1	1
Philippines	52.9	7,190	14.9	112	1.6	9	2	2	4,088	8.8	125	1	9.9	3	3
Singapore	2.9	429	7.7	72	0.8	4	8	7	208	3.8	61	0.4	3.6	9	2
Thailand	35.5	8,622	16.2	126	1.7	10	2	2	5,015	9	129	1	10.1	4	2
Timor-Leste	0.7	50	12.5	93	1.4	14	2	2	24	6.2	89	0.7	10.8	3	3
Viet Nam	48.7	4,177	7.1	54	0.8	6	7	3	2,420	4	58	0.5	5.5	6	7
**South Central Asia**															
Afghanistan	17.6	694	6.6	50	0.7	7	2	2	520	5.3	80	0.6	7.2	4	2
Bangladesh	82.5	8,068	10.6	78	1.2	12	2	2	5,214	7.1	103	0.8	11.5	3	2
Bhutan	0.4	48	14.4	105	1.6	18	1	1	32	10.2	146	1.2	14.9	2	1
India	652.5	96,922	14.7	111	1.6	17	2	2	60,078	9.2	133	1	16.3	2	2
Iran, Islamic Rep.	40.8	917	2.2	16	0.2	2	16	10	467	1.2	16	0.1	2	12	10
Kazakhstan	9.5	1,729	15.7	119	1.6	10	2	2	838	7.5	106	0.8	8.4	4	1
Kyrgyzstan	3.1	601	19.9	153	2.1	17	2	1	318	10.9	159	1.2	15.1	1	1
Maldives	0.2	41	23.2	167	2.6	19	2	2	21	13.4	178	1.7	24.7	2	2
Nepal	15.2	2,942	21.5	161	2.3	19	1	1	1,928	14.3	209	1.6	17.3	2	1
Pakistan	97.7	5,601	7.3	54	0.8	6	3	2	3,861	5.2	75	0.6	6.8	3	3
Sri Lanka	10.9	1,136	7.8	63	0.9	9	2	4	643	4.2	64	0.5	9.4	2	3
Tajikistan	4.5	220	5.7	45	0.6	8	5	2	116	3.2	49	0.3	6.2	4	2
Turkmenistan	3	397	13.6	105	1.4	13	2	2	247	8.8	130	1	12	2	2
Uzbekistan	16.2	1,608	9.9	75	1.1	11	2	2	850	5.4	79	0.6	9.9	3	2
**Western Asia**															
Armenia	1.6	196	8.4	69	1.0	5	8	2	136	5.6	83	0.7	4.8	7	2
Azerbaijan	5	397	6.5	50	0.7	7	4	2	272	4.6	65	0.6	7.5	4	2
Bahrain	0.6	19	3.8	25	0.5	3	8	6	12	2.7	34	0.4	4.1	6	8
Georgia	2	297	9.8	75	1.0	7	4	2	186	5.5	78	0.6	7.2	4	2
Iraq	19.4	244	1.9	13	0.2	2	13	10	159	1.3	18	0.1	2.2	12	9

Country	Total female population*	Nb of cases	ASIR	SIR	CIR (%)	% of all cancers	Rank (all ages)	Rank (15-44y)	Nb of deaths	ASMR	SMR	CIR (%)	% of all cancers	Rank (all ages)	Rank (15-44y)
Israel	4.3	241	4.8	37	0.5	2	14	5	132	2.1	35	0.2	2.2	13	5
Jordan	4.9	104	2.9	20	0.3	2	12	10	61	1.8	25	0.2	2.3	14	7
Kuwait	1.8	59	3.3	23	0.4	3	8	3	31	2.2	27	0.3	3.9	8	4
Lebanon	3	192	5.7	46	0.6	2	10	7	125	3.6	56	0.4	3.2	9	7
Oman	1.6	77	6.3	44	0.7	6	4	4	41	3.9	51	0.5	6.9	4	4
Palestine	2.5	38	2.5	17	0.3	2	16	11	27	1.9	26	0.2	2.2	13	10
Qatar	0.7	19	4.0	23	0.5	4	5	5	12	3.2	35	0.5	5.2	5	4
Saudi Arabia	14.3	316	2.5	18	0.3	3	9	9	158	1.5	19	0.2	3.6	9	6
Syrian Arab Republic	9.1	259	3.5	27	0.4	2	12	9	190	2.7	39	0.3	2.7	9	8
Turkey	41.5	2,356	4.8	37	0.5	3	13	5	1,280	2.5	37	0.3	3.1	10	8
United Arab Emirates	2.7	108	6.4	31	0.7	4	5	4	56	4.4	38	0.5	5.8	5	4
Yemen	14.3	170	1.9	14	0.2	2	13	8	115	1.4	20	0.2	2.4	12	10
**Eastern Europe**															
Belarus	5.1	979	13.3	95	1.3	5	9	3	318	3.8	51	0.4	3.9	10	2
Bulgaria	3.6	1,080	20.3	138	2.0	7	4	2	475	7.4	96	0.8	6	5	1
Czech Republic	5.4	813	9.9	72	1.0	3	12	3	435	4	63	0.4	3.6	8	2
Hungary	5.1	1,312	17.2	122	1.7	4	6	2	499	5.1	75	0.6	3.3	8	2
Moldova	2.1	639	21.4	158	2.2	9	3	1	259	7.9	112	0.9	7.4	4	1
Poland	19.7	3,220	9.4	80	1.0	4	7	4	1,947	4.9	79	0.6	4	9	3
Romania	10.1	3,308	19.5	158	2.2	9	3	2	1,743	8.9	135	1	8.4	4	2
Russian Federation	77.1	18,164	17.0	117	1.6	7	4	2	7,579	6.2	81	0.6	5.3	8	1
Slovakia	2.8	692	16.6	122	1.7	5	6	2	281	5.7	83	0.6	4.1	9	1
Ukraine	23.7	5,733	17.0	116	1.7	7	4	2	2,475	6.6	82	0.7	5.7	9	1
**Northern Europe**															
Denmark	2.9	415	10.9	72	1.0	2	11	3	131	2	37	0.2	1.6	15	3
Estonia	0.7	230	22.5	159	2.2	7	5	2	60	4.3	65	0.5	3.3	10	2
Finland	2.8	182	4.7	32	0.4	1	18	4	64	0.9	18	0.1	1.1	19	5
Iceland	0.2	15	7.6	50	0.6	2	13	2	3	1.3	17	0.2	1	17	4
Ireland	2.4	340	11.0	77	1.0	3	9	4	107	2.9	42	0.3	2.3	14	2

Country	Total female population*	Nb of cases	ASIR	SIR	CIR (%)	% of all cancers	Rank (all ages)	Rank (15-44y)	Nb of deaths	ASMR	SMR	CIR (%)	% of all cancers	Rank (all ages)	Rank (15-44y)
Latvia	1	339	25.0	151	2.3	6	5	1	134	6.5	93	0.7	4.7	9	2
Lithuania	1.6	431	18.9	131	1.9	5	4	2	209	7.2	100	0.8	5.6	7	1
Norway	2.6	361	10.7	72	1.0	3	10	3	89	1.7	29	0.2	1.7	16	4
Sweden	5	558	9.0	58	0.8	2	11	3	222	2	37	0.2	2	14	2
United Kingdom	33.7	3,430	8.4	52	0.7	2	13	2	1,033	1.7	26	0.2	1.2	18	2
**Southern Europe**															
Albania	1.5	134	6.5	50	0.7	4	8	4	53	2.3	34	0.3	3.4	10	6
Bosnia Herzegovina	1.8	556	23.9	152	2.2	9	3	1	141	4.7	63	0.5	3.9	10	2
Croatia	2.2	266	7.9	59	0.8	2	13	3	175	3.7	61	0.4	2.8	12	2
Cyprus	0.6	45	5.7	42	0.5	2	12	3	18	1.5	29	0.1	2	14	3
FYR Macedonia	1	151	10.0	76	1.0	4	6	3	59	3.5	51	0.4	3.8	11	5
Greece	5.7	696	8.1	57	0.8	3	12	4	271	2.1	36	0.2	2.1	11	3
Italy	30.4	3,105	7.1	46	0.7	2	16	4	986	1.5	23	0.2	1.3	17	4
Malta	0.2	11	3.5	25	0.4	1	17	6	7	1.4	26	0.1	1.8	16	28
Montenegro	0.3	54	12.5	89	1.2	5	4	2	21	4.2	58	0.4	3.9	7	2
Portugal	5.4	750	8.9	64	0.8	3	9	3	340	2.8	46	0.3	3	13	3
Serbia	4.5	1,327	20.3	149	2.1	6	5	2	551	7	101	0.8	4.8	6	2
Slovenia	1	110	7.1	50	0.7	2	14	3	65	2.8	47	0.3	2.3	13	2
Spain	23.6	1,942	5.2	38	0.5	2	16	4	825	1.7	26	0.2	1.9	16	5
**Western Europe**															
Austria	4.5	390	5.5	42	0.5	2	14	4	163	1.7	28	0.2	1.6	17	3
Belgium	5.8	640	7.8	56	0.8	2	14	4	235	2	33	0.2	1.8	17	5
France	33.1	3,067	6.7	47	0.6	2	13	4	1,472	2.3	36	0.2	1.9	16	4
Germany	41.7	4,608	7.5	51	0.7	2	15	4	2,011	2.2	35	0.2	1.8	17	3
Luxembourg	0.3	25	5.6	45	0.5	2	12	4	11	2	33	0.2	2.2	13	2
Switzerland	4.3	258	3.8	29	0.4	1	18	6	104	1.1	19	0.1	1.3	19	7
The Netherlands	8.6	670	5.7	39	0.5	1	12	3	250	1.4	23	0.1	1.1	19	4
**Australia/New Zealand**															

Country	Total female population*	Nb of cases	ASIR	SIR	CIR (%)	% of all cancers	Rank (all ages)	Rank (15-44y)	Nb of deaths	ASMR	SMR	CIR (%)	% of all cancers	Rank (all ages)	Rank (15-44y)
Australia	12.4	924	6.0	41	0.5	1	14	5	331	1.7	24	0.2	1.6	18	3
New Zealand	2.4	190	6.0	43	0.6	2	13	4	72	1.8	27	0.2	1.6	18	2
**Melanesia**															
Fiji	0.4	124	25.9	198	2.6	14	2	2	94	19.7	286	2.1	18.5	2	2
New Caledonia	0.1	30	16.5	132	1.5	5	5	3	16	8.2	127	0.8	8.8	3	2
Papua New Guinea	4.1	1,024	29.1	232	2.7	16	2	2	663	19.8	305	2	17.5	2	1
Solomon Islands	0.3	55	22.6	183	2.3	18	2	2	39	16	272	1.6	22.7	1	1
Vanuatu	0.1	21	17.0	138	1.7	18	2	2	13	10.6	173	1.1	19.7	2	1
**Micronesia/Polynesia**															
Guam	0.1	18	18.7	142	2.1	10	3	2	9	8.1	127	1	8.2	3	4
French Polynesia	0.1	17	10.1	78	1.1	5	6	3	7	4.3	60	0.5	4	6	4
Samoa	0.1	10	12.6	93	1.5	5	5	3	6	7.5	103	1	6	5	5

ASIR: age-standardised incidence rate; ASMR: age-standardised mortality rate; SIR: standardised incidence ratio; SMR: standardised mortality ratio; CIR: cumulative incidence rate; CMR: cumulative mortality rate; DR: Democratic Republic; DPR: Democratic People's Republic; PDR: People's Democratic Republic; Rep: Republic; HDI: human development index: 1=very high; 2=high; 3=medium; 4=low..
* in millions; † all cancers except non-melanoma cancers; ‡ Eswatini: previously named Swaziland.

STROBE Statement—checklist of items that should be included in reports of observational studies (von Elm, *Ann Intern Med* 2007; 147: 573-577)

	Item No	Recommendation	Implementation of STROBE items
**Title and abstract**	1	(*a*) Indicate the study's design with a commonly used term in the title or the abstract	Title makes clear that design is descriptive analysis of the burden of cervical cancer in 2018
		(*b*) Provide in the abstract an informative and balanced summary of what was done and what was found	A balanced and informative summary is provided in the abstract structured according to Lancet instructions.
**Introduction**			
Background/rationale	2	Explain the scientific background and rationale for the investigation being reported	See first four paragraphs of the Introduction.
Objectives	3	State specific objectives, including any prespecified hypotheses	See last sentence of the Introduction.
**Methods**			
Study design	4	Present key elements of study design early in the paper	Observational study using standard components of recent cancer and mortality registries completed with extrapolations towards 2018 and extrapolations for countries with incomplete or lacking data using the best available representative data sources.
Setting	5	Describe the setting, locations, and relevant dates, including periods of recruitment, exposure, follow-up, and data collection	The geographical area are 185 countries, aggregated at subcontinent and world level. Estimates concern the year 2018.
Participants	6	(*a*) *Cohort study*—Give the eligibility criteria, and the sources and methods of selection of participants. Describe methods of follow-up<br>*Case-control study*—Give the eligibility criteria, and the sources and methods of case ascertainment and control selection. Give the rationale for the choice of cases and controls<br>*Cross-sectional study*—Give the eligibility criteria, and the sources and methods of selection of participants	*Study population:*<br>Whole female population grouped by 5-year age group, with oldest group consisting of women aged 85 and older, aggregated by country, subcontinent, categories of human development index (HDI) as defined by UN Development Programme and whole world.
		(*b*) *Cohort study*—For matched studies, give matching criteria and number of exposed and unexposed	

	Item No	Recommendation	Implementation of STROBE items
		Case-control study—For matched studies, give matching criteria and the number of controls per case	
Variables	7	Clearly define all outcomes, exposures, predictors, potential confounders, and effect modifiers. Give diagnostic criteria, if applicable	Outcomes: number of cases and deaths from cervical cancer, age-standardised incidence and mortality rates, standardised incidence and mortality ratios, cumulative incidence or mortality rates, ranking of cervical cancer incidence and mortality among all cancers, proportional incidence and mortality.
Data sources/ measurement	8*	For each variable of interest, give sources of data and details of methods of assessment (measurement). Describe comparability of assessment methods if there is more than one group	The main sources of data are the most recent available national or regional cancer registries, WHO mortality database and UN population data that were compiled in the GLOBOCAN database.
Bias	9	Describe any efforts to address potential sources of bias	Indirect and indirect standardisation were used to control for different age compositions of population. Diverse extrapolation methods (explained §2 & 3) were applied to compute incidence and mortality from countries for which data were incomplete or of insufficient quality.
Study size	10	Explain how the study size was arrived at	Not of application
Quantitative variables	11	Explain how quantitative variables were handled in the analyses. If applicable, describe which groupings were chosen and why	Groupings by country, subcontinent and whole world, separate grouping for young women aged 15-44 years, grouping by human development index.
Statistical methods	12	(*a*) Describe all statistical methods, including those used to control for confounding	Direct and indirect standardisation to control confounding by age. Geographical distribution of the standardised incidence and mortality rates by country is displayed in choropleth world maps and bar charts by continent and country, sorted by decreasing age-standardised mortality.
		(*b*) Describe any methods used to examine subgroups and interactions	

	Item No	Recommendation	Implementation of STROBE items
		(*c*) Explain how missing data were addressed	A hierarchy of methods was used that were dependent on the availability and quality of the source information from population-based cancer registries; methods ranged from a short-term extrapolation of high quality recorded national incidence rates via short-term prediction models[19] through to the use of observed rates from one or more neighbouring countries in the same region in the complete absence of recorded data.
		(*d*) *Cohort study*—If applicable, explain how loss to follow-up was addressed<br>*Case-control study*—If applicable, explain how matching of cases and controls was addressed<br>*Cross-sectional study*—If applicable, describe analytical methods taking account of sampling strategy	Not of application.
		(*e*) Describe any sensitivity analyses	Not of application.
**Results**			
Participants	13*	(a) Report numbers of individuals at each stage of study—eg numbers potentially eligible, examined for eligibility, confirmed eligible, included in the study, completing follow-up, and analysed	The whole female population of 185 countries was included.
		(b) Give reasons for non-participation at each stage	Not of application (see 13a). Countries that were not recognised by the United Nations were excluded.
		(c) Consider use of a flow diagram	Not of application.
Descriptive data	14*	(a) Give characteristics of study participants (eg demographic, clinical, social) and information on exposures and potential confounders	Whole populations were characterised by their age composition and socio-economic development level (HDI). The distribution by HDI is displayed in choropleth map in Suppl Figure 1. The quality of data sources to estimate quality of incidence and mortality by country is described in choropleth maps (Suppl Figures 4 and 5).

	Item No	Recommendation	Implementation of STROBE items
		(b) Indicate number of participants with missing data for each variable of interest	The quality of data sources to estimate quality of incidence and mortality by country is described in choropleth maps (Suppl Figures 4 and 5). These maps identify the countries with missing data for which data had to be estimated from representative neighbouring countries.
		(c) *Cohort study*—Summarise follow-up time (eg, average and total amount)	Not of application.
Outcome data	15*	*Cohort study*—Report numbers of outcome events or summary measures over time	The number of estimated outcome events are included in Table 1 for the whole world, by HDI level and by subcontinent, and in Suppl Table 1 by country.
		Case-control study—Report numbers in each exposure category, or summary measures of exposure	
		Cross-sectional study—Report numbers of outcome events or summary measures	
Main results	16	(*a*) Give unadjusted estimates and, if applicable, confounder-adjusted estimates and their precision (eg, 95% confidence interval). Make clear which confounders were adjusted for and why they were included	Diverse incidence and mortality indicators are presented in structured tables, choropleth maps, and sorted bar charts. (Tables 1, Figures 1-3; Suppl Tables 1).
		(*b*) Report category boundaries when continuous variables were categorized	The distribution of age-standardised incidence and mortality by HDI level (very high, high, moderate and low) is shown in the boxplots in Suppl Figure 2.
		(*c*) If relevant, consider translating estimates of relative risk into absolute risk for a meaningful time period	Absolute standardised rates and cumulative risks up to the age of 74 risks as well as standardised relative incidence and mortality ratios are listed in Table 1 and Suppl Table 1.
Other analyses	17	Report other analyses done—eg analyses of subgroups and interactions, and sensitivity analyses	The correlation between age-standardised incidence and the average high-risk HPV prevalence, derived from a recent meta-analysis in show in an
**Discussion**			
Key results	18	Summarise key results with reference to study objectives	Key results are summarised in the first § of the Discussion.
Limitations	19	Discuss limitations of the study, taking into account sources of potential bias or	Limitations are discussed in the section “data quality” of the

	Item No	Recommendation	Implementation of STROBE items
		imprecision. Discuss both direction and magnitude of any potential bias	Discussion. Only 24% and 44% of countries provided directly usable national incidence and mortality data, respectively. For 32 and 84 countries, no information could be identified for incidence or mortality, respectively, and estimates have been computed either from modelling or from neighbouring countries. The problems related to the death cause coded as uterine cancer not otherwise specified, where it is unclear whether the cancer originates from the cervix uteri or from the corpus uteri is discussed.<br><br>The successive GLOBOCAN publications present an estimate of the world-wide burden of cancers using the best available data and extrapolation methods for a given year but are not a good base for time trend analyses. To assess impact of interventions, it is recommended to use long-term time series from selected high-quality registries.
Interpretation	20	Give a cautious overall interpretation of results considering objectives, limitations, multiplicity of analyses, results from similar studies, and other relevant evidence	The Discussion highlights that the reliability of incidence and mortality estimates is determined by the quality and completeness of registration, and by the appropriateness of external data used to derive unavailable data. The GLOBOCAN 2018 estimates should be compared with future publications of observed cancer incidence & mortality data from national registries for the year 2018.
Generalisability	21	Discuss the generalisability (external validity) of the study results	In spite of recognised limitations, the presented estimates of the current burden of cervical cancer in the world can be considered as the best possible given present available data.

	Item No	Recommendation	Implementation of STROBE items
			This 2018 burden paper may serve as a baseline for the targets of the global strategy.
**Other information**			
Funding	22	Give the source of funding and the role of the funders for the present study and, if applicable, for the original study on which the present article is based	Sources of funding are mentioned in the aacknowledgements section at the end of the manuscript. No funding bodies had any role in study design, data collection and analysis, decision to publish, or preparation of the manuscript.
